# Exploring Women Healthcare Leaders' Perceptions on Barriers to Leadership in Greek Context

**DOI:** 10.3389/fpubh.2019.00068

**Published:** 2019-04-09

**Authors:** Stavroula Kalaitzi, K. L. Cheung, M. Hiligsmann, S. Babich, K. Czabanowska

**Affiliations:** ^1^Department of International Health, Faculty of Health, Medicine and Life Sciences, Care and Public Health Research Institute, Maastricht University, Maastricht, Netherlands; ^2^Department of Health Promotion, Faculty of Health, Medicine and Life Sciences, Care and Public Health Research Institute, Maastricht University, Maastricht, Netherlands; ^3^Department of Clinical Sciences, College of Health and Life Sciences, Brunel University, London, United Kingdom; ^4^Department of Health Services Research, Faculty of Health, Medicine and Life Sciences, Care and Public Health Research Institute, Maastricht University, Maastricht, Netherlands; ^5^Richard M. Fairbanks School of Public Health, Indiana University, Indianapolis, IN, United States; ^6^Department of Health Policy and Management, Faculty of Health Sciences, Institute of Public Health, Jagellonian University, Kraków, Poland

**Keywords:** women's leadership, barriers, healthcare, perceptions, Greece, best worst scaling, online questionnaire, economic crisis

## Abstract

**Background:** Gender inequalities have been identified as important derailment factors for health workforce and health system sustainability. Literature holds responsible a list of gendered barriers faced by female health workforce. However, there is a gap in the evidence based research on women leaders' own perceptions of barriers to leading positions advancement. This study aims to explore leadership barriers perceived by women healthcare leaders within country's context; research focused on Greece due to country's poor performance on gender equality index and current economic turbulence. Study supplements survey data and provides orientation for further gender sensitive research in health workforce development through country's specificity lens to better inform education and policy makers.

**Methods:** The best-worst object case survey method was used, applying an online questionnaire designed in Qualtrics. The online questionnaire was sent to 30 purposively invited participants. Respondents were asked to tick the most and the least important barriers to women's leadership in provided choice scenarios. Descriptive data analysis was used to understand and interpret the results.

**Results:** Women leaders perceived stereotypes, work/life balance, lack of equal career advancement, lack of confidence, gender gap and gender bias to be the barriers with the greatest relative importance in constraining opportunities for pursuing leading positions in Greek healthcare setting. Twenty more barriers were identified and ranked lower in relative importance. The results are considered exploratory and not to obtain population based outcomes.

**Conclusion:** This exploratory study reports the perceived barriers of women leaders in pursuing leading positions within Greek healthcare context. The findings point mainly to organizational and socio-cultural related barriers potentially aggravated by country's unfortunate current economic turbulence. Further extensive research is required to establish grounded conclusions and better inform education and policy makers in developing gender sensitive strategies to sustainable health workforce development.

## Introduction

Gender inequalities in the global healthcare workforce have been identified as important derailment factors for health workforce and health systems' sustainability. The healthcare sector is a steadily increasing source of employment in most OECD countries with women representing the vast majority of the specialized health workforce (1). Hence, it would be reasonable to expect a powerful influential women presence in health and healthcare decision making circles and especially across the spectrum of clinical practice, education, planning, advocacy, and policy. However, in spite of the fact that 75% of the global healthcare workforce is comprised of women in some countries, only about 25% of those women hold leadership positions (2). In the healthcare provision sector, women leaders represent only 18% of hospital CEOs and 14% of healthcare boards of directors (3); in clinical leadership, only15.9% of women have reached top level positions (4); in academic medicine, Grade A has been achieved only by 14% of women pursuing a high-level career in the field (5).

The added value of women's leadership in health and healthcare has been addressed extensively by literature (3, 4, 6–11); the excellent qualities and results to health systems outcomes both at universal health coverage and at national and community level have been evidenced extensively; the importance of gender equality and diversity of health workforce have also been acknowledged by scholarship and global agencies, such as WHO (12), OECD (1), as a governance priority to strengthen health services, professional education and employment systems and make health systems responsive to life events and societal challenges.

Global health organizations, such as WHO, argue that the health sector is a good place to start unlocking the full potential of women at work and achieving progress toward meeting the United Nations Sustainable Development Goals (SDGs) (12). The relationship between gender (SDG5 and in particular to SDG5.5 “Ensure women's full and effective participation and equal opportunities for leadership at all levels of decision-making”) and health (SDG3) and their intersection across multiple SDGs, such as SDG 8 (gender and the health workforce, formal and informal, decent work, fair employment), SDG4 (education), SDG10 & 17 (accessible services), SDG16 & 17 (governance) and SDG1 & 8 & 9 (macroeconomic policies), accentuate the catalyzing role of gender equality and diversity toward achieving progress at all levels, interpersonal, institutional, societal, national, and global (13). In line with this rationale, European Union through its constitutional bodies, such as European Commission and its agencies and European Parliament, address health as a core issue interconnecting well-being of individuals and societies, social inclusion, economic growth, and environmental protection (14). It was also explicitly acknowledged that health and healthcare systems are tightly linked to social and employment policies where gender inequalities, such as work life balance, employment contracts, are integral part of well-functioning societies and economies and should be counted in the equation for achieving inclusive growth in the twenty-first century societies (15).

On the other hand, gender asymmetries are considered a universal fact of human societies reflecting the distinction between power and culturally legitimized authority, the ability to gain compliance and recognition “taking male authority for granted and accepting somehow the exercise of power by women as not that important or secondary to their expected social role” (16, 17). The hierarchical gender stratification of careers may be considered that are supported by hierarchical relations of women and men in society (18); example given in healthcare sector where in spite of increased feminization of health workforce, women remain severely underrepresented in leading positions.

Multiple studies have explored the journey of women leadership in healthcare dissecting, among others, working patterns, styles, roles, institutional processes, sector insufficiencies, governance flaws. However, little attention has been drawn on women leaders' perceptions on barriers constraining their increased presence in healthcare leading roles which is not mirrored accordingly in respective leading roles. Leadership requires several qualities and healthcare leadership is no exception, given the complex, unprecedented challenges healthcare systems and societies are currently facing (19, 20). The leadership prism reveals itself differently in each context and culture (21); it is largely shaped by context, gender and culture, reflecting dynamic relationships among its components (22). Cultural and socioeconomic contexts, oftentimes intensified by unfortunate economic or social turbulences, influence the socially accepted perceptions on legitimized gendered authority and leadership both at societal and professional level (16, 23); the deeply rooted process of durability and transferability of these perceptions may intercept the course of change needed in modern societies.

The aim of this paper is to provide the findings of a small exploratory study sought to discover the perceptions of women leaders on perceived barriers to women leadership advancement within country's healthcare context; research focused on Greece due to country's poor performance on gender equality index and current economic turbulence. Study supplements survey data which provides orientation for further gender sensitive research in health workforce development through country's specificity lens to better inform education and policy makers.

## Methods

### Country

Greece was of interest as the survey target because it was recently announced in European Institute of Gender Equality progress report (24) that descended to the lowest rank in the Gender Equality Index (50.0), being the only EU country with a deteriorating score over a 10-year period in the domain of economic and social power of women. Greece was also ranked low in women's representation in the medical workforce among OECD countries; out of 65,499 doctors, 27,549 were women, namely 41.20% (25, 26), whereas it is estimated that only 11% assume academic professorship (27). In addition, the Greek healthcare system was profoundly affected by the recent financial and debt crisis of 2009 suffering, amongst others, from several inefficiencies, health workforce included (28); dramatic salary cuts, non-renewal of employment contracts resulting in under-staffing; unofficial expectations for long, unpaid work hours, long unemployment periods, or employment on part–time basis deteriorated significantly key indicators on employment and population health (29). The crisis affected predominantly women and single-parent families (30, 31). Austerity intensified discrimination against women, especially in employment forms and under-payment (32) and supported re-establishment of stereotypes mainly in health services sector, social care, education, and public administration. Any progress gained in the field of gender equality and equal work opportunities during 1980s was compromised generating a backlash in employment practices and choices (30, 31).

### Participants

The stratified sampling technique across academic, clinical, and medical settings was applied to identify the important common patterns or variations cutting across healthcare settings and to gain an understanding of perceptions of gendered barriers to women's leadership across Greek healthcare settings (33). Researchers aimed to recruit 20–30 women leaders as participants representing an appropriate cross sectors variation sample in a typical case sampling for exploring perceptions (20, 34); the identified sample size and sampling method was deemed by the authors as the most applicable for the study's purposes (33, 35).

Participants were identified through publicly announced women leaders' email addresses found via a systematic web search of Greek healthcare organizations. Snowball sampling technique was also applied as better combined with sampling strategy for examination of commonalities and differences (33). First, medical, nursing, and public health schools were identified in the web site of Ministry of Education and then separately explored. Next, all hospitals presented in the web site of the Ministry of Health were identified and separately searched. Finally, medical and health organizations and associations were also identified through the web site of the Ministry of Health and separately searched.

The following inclusion criteria were used to retrieve, select and accept women leaders' email addresses across healthcare settings: (a) Academic setting (medical/nursing schools, public health school): full/assistant professor; (b) Clinical setting (public/private hospital): CEO, vice president, board member, clinical director, assistant clinic director; (c) Medical setting (medical/health body, health ministry): president, vice president, board member, director, assistant director. Contact information of invited participants was collected between April 2017–August 2017. Men healthcare leaders were excluded as not being within the scope of this small exploratory study; researchers acknowledge the researched women's perspectives to be initial and exploratory and further gender balanced research is required to yield grounded conclusions.

Participants were assured anonymity throughout the survey procedure since online questionnaires were anonymously replied and registered in Qualtrics (36). Invited participants were informed about the study's purpose, procedure, anonymity, and their rights via the invited introductory email message. Data are stored to university's server and are protected via password owned by involved researchers only.

### Online Questionnaire Instrument

The Best Worst Scaling (BWS) method was used to identify the most and least important barriers across the identified three healthcare settings. Best-Worst Scaling was deemed appropriate for this exploratory study since it has been increasingly used to investigate preferences over a number of topics in the healthcare field (37). It was also considered the best choice for this study, as ranking tasks are simplified and it facilitates the evaluation of varying degrees of different barriers involved in composite decisions (38). It also normalizes all relative–importance weights to the (0,1) interval and, thus, eliminates scale artifacts and reduces social desirability bias, since respondents evaluate trade-offs between attributes (39).

The online questionnaire was designed using the Sawtooth Software (40). Four different versions of a self-administered questionnaire and an explanatory introduction were developed. One open-ended question was included, providing participants the possibility to mention additional barriers or to fill in freehand comments. Demographic and professional features (gender, age, professional role) were considered at the beginning. Each participant was asked to describe their professional role, selecting one out of three different options: academic, clinical and medical, as defined for this study.

**Figure 1:**
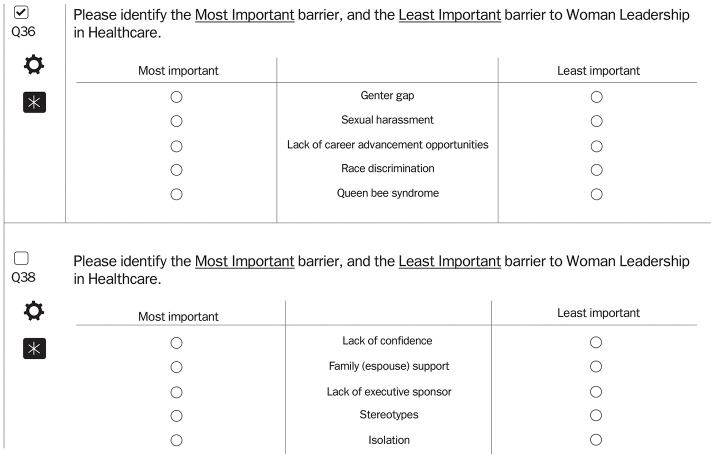
The BWS choice scenarios addressed to participants.

A total of 14 choice scenarios were presented including a set of five barriers with varying combinations and ordering of barriers. Four versions of 14 selected choice scenarios were developed and each respondent received randomly one of the four versions. A snapshot from the choice scenarios addressed to participants is presented in Figure 1.

At the end of the questionnaire, participants were asked to rate the difficulty of completing the choice scenarios based on a Likert scale (1 = very easy, to 7 = very difficult) (41). The BWS survey was sent to participants via Qualtrics on January 16, 2018 and closed on February 6, 2018. It was active for an initial period of 2 weeks, and a reminder was sent out on January 30, 2018.

### Identification of Barriers

Participants were asked to identify the most and least preferred barriers from the choice scenario list of five barriers based on the Barriers Thematic Map (BTM) (42). The BTM was deemed appropriate for this exploratory study since it reports a comprehensive list of 26 barriers to women leadership with varying degrees of prevalence (Table 1) compiled using a multi-method approach and validated from several experts and focus groups during dedicated workshops (42, 43).

**Table 1:** Barriers Thematic Map (BTM) to women's leadership.

**Barriers thematic map (BTM) to women's leadership**	
1	Age
2	Lack of (equal) career advancement opportunities
3	Culture
4	Lack of family (espousal) support
5	Gender bias (discrimination)
6	Gender gap
7	Gender pay gap
8	Glass ceiling
9	Glass cliff
10	Isolation
11	Lack of executive sponsor
12	Lack of flexible working environment
13	Lack of confidence
14	Lack of mentoring
15	Lack of networking
16	Lack of leadership skills
17	Personal health
18	Queen bee syndrome
19	Race discrimination
20	Lack of role model
21	Sexual harassment
22	Lack of social support
23	Stereotypes
24	Limited succession planning
25	Tokenism
26	Work/life balance

### Approach to Analysis

All fully answered online questionnaires were deemed completed and included in the data analysis. The calculation of the mean relative importance score (RIS) with its 95% confidence interval, generated by the Hierarchical Bayes (HB) estimation using Sawtooth platform, allowed for ranking the barriers from the most to least important (44, 45).

## Results

### Participant Characteristics

A total of thirty online questionnaires were sent out; twenty-four participants completed the online questionnaire and were included in the analysis; their basic demographics are summarized in Table 2. The responsive rate of 80% (24/30) calculated by dividing the number of usable responses returned by the invited participants' number (24/30) and was deemed appropriate and supported this exploratory study's findings (35, 46–48).

**Table 2:** Demographics of participants.

**Participant characteristics**	**Percentage (*n* = 24)**
**PROFESSIONAL ROLE**	
Academic	12 (50%)
Clinical	11 (46%)
Medical	1 (4%)
**AGE**	
39–50	11 (46%)
51–60	10 (42%)
61–65	3 (13%)
**DIFFICULTY TO REPLY (7-POINT LIKERT SCALE)**	
1–3 (less difficult)	12 (50%)
4–5 (medium difficult)	10 (42%)
6–7 (very difficult)	2 (8%)

All participants were women; respondents assumed mostly academic and clinical leading roles (50 and 46%, respectively). The age of the respondents ranged from 39 to 50 years (46%, *n* = 11), 51–60 years (42%, *n* = 10), 61–65 years (13%, *n* = 3) representing mainly mid-career stage, namely the entryway to senior leading positions. All respondents had an overall fit statistic higher than 0.25 and were thus all included in the analysis (40). Respondents rated BWS survey as less to medially difficult on a 7-point Likert scale rates ranging from 2 to 6 (mean:3.45) (41). Five comments were provided in the open-ended question field related to barriers focusing mostly on lack of organizational support and socially and culturally related barriers.

### Relative Importance of Barriers to Women's Leadership in Healthcare in Greece

The RIS (Relative Importance Score) of the barriers is illustrated in Table 3.

**Table 3:** Barriers to women's leadership in Greek healthcare and their Relative Importance Scores (RIS) based on Hierarchical Bayes (HB) estimation.

**Barriers to women's leadership in Greek healthcare and their relative importance scores (RIS)**	
Stereotypes	8.80 (7.06–10.55)
Work/life balance	6.22 (4.24–8.20)
Lack of equal career advancement opportunities	5.72 (4.26–7.18)
Lack of confidence	5.25 (3.32–7.18)
Gender gap	5.25 (3.25–7.25)
Gender bias	5.18 (3.55–6.81)
Glass ceiling	4.72 (2.87–6.57)
Lack of family (espouse) support	4.71 (3.69–5.73)
Lack of role models	4.70 (3.31–6.09)
Lack of social support	4.46 (3.44–5.48)
Lack of flexible working environment	4.36 (2.64–6.07)
Lack of leadership skills	4.21 (2.82–5.59)
Lack of networking	4.01 (2.83–5.19)
Lack of mentoring	3.79 (2.40–5.18)
Isolation	3.22 (1.61–4.82)
Culture	3.21 (2.08–4.34)
Limited succession planning	3.09 (2.49–3.69)
Glass cliff	2.64 (1.66–3.62)
Personal health	2.48 (1.18–3.78)
Gender pay gap	2.34 (1.63–3.05)
Queen bee syndrome	2.07 (1.13–3.02)
Tokenism	1.97 (0.98–2.97)
Lack of executive sponsor	1.97 (0.94–2.99)
Race discrimination	1.93 (1.30–2.55)
Sexual harassment	1.89 (1.45–2.33)
Age	1.68 (0.88–2.48)

**Figure 2:**
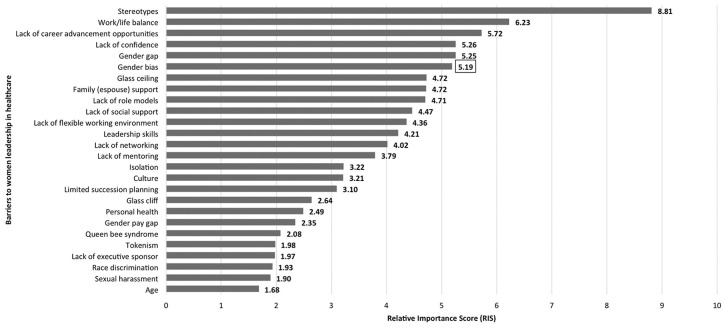
Ranked barriers to women's leadership in healthcare in Greece (*n* = 24). The RIS in box indicates the most important barriers' threshold (RIS > 5.19).

Figure 2 shows ranking of barriers to women's leadership in Greek healthcare context; the visual cut-off point (RIS = 5.19) may be considered as a threshold to differentiate the most important barriers from the remaining ones in this study.

Out of twenty-six, the six most important barriers (RIS > 5.00) to women's leadership in Greek healthcare settings (*n* = 24) included stereotypes (RIS:8.80), work/life balance (RIS:6.22), lack of equal career advancement opportunities (RIS:5.72), lack of confidence (RIS:5.25), gender gap (RIS:5.25), and gender bias (RIS:5.19). Stereotypes tops the barriers relative importance list (RIS:8.80) with considerable distance from the second item, “work/life balance” (RIS:6.22), whereas “lack of equal career opportunities,” “lack of confidence,” “gender gap,” and “gender bias” share ranking in the vicinity of RIS:5.72-5.19. The two most targeted barriers, namely stereotypes and work/life balance, may indicate some relatedness or even complementarity between them in fostering and maintaining socially deeply rooted gendered roles within country's specific context. Organizational and socio-cultural bounded barriers were the main concerns of respondents; four out of the five responses to open ended question of the online questionnaire corroborate the findings on lack of organizational support and cultural constraints to be the harshest constraints to be dealt with.

Medium relative importance barriers include fifteen barriers ranging from RIS:4.72–2.07 describing challenges both at personal and at organizational level, such as lack of family (espousal) support, lack of mentoring, lack of leadership skills and glass ceiling, glass cliff, lack of flexible working environment. However, nonetheless their abundance and variety the medium relative importance ranking may be indicate that are not perceived too rigid or unsurmountable.

Five out of the 26 barriers have been reported in the lowest relative importance ranks raging from RIS 1.97–1.68 and include “tokenism,” “lack of executive sponsor,” “race discrimination,” “sexual harassment,” and “age.” However, underlying dynamic relations among organizations' structure, society, and country's economic turbulence may have influenced the attention drawn to these constraints of which the research importance should not be underestimated.

## Discussion

The importance of organizational and socio-cultural contexts in developing and fostering barriers to women's leadership in Greek healthcare setting was emerged from respondents' replies. Participants perceived stereotypes, work/life balance, lack of equal career advancement, lack of confidence, gender gap, and gender bias to be the barriers with the greatest relative importance. These barriers may have direct effects in discouraging women to pursuit leading roles in medical practice, education, and medical organizations. Skills and talent may be wasted; gender diversity and inclusion efforts within healthcare organizations may be compromised initiating cascading effects on organizational culture and performance.

In line with literature, this small exploratory study's findings point to a mix of perceived barriers which may elucidate women's poor promotion and retention from leadership positions in healthcare. Downs et al. (8) argue that elevating women neutralizes gender equality threat and create a ripple effect benefiting families, communities, organizations, and countries. Newman (4) asserts gender discrimination and inequalities impede the development of robust workforces resulting in critical systems inefficiencies; hence, gender balanced health workforce should be a leadership and governance priority both in education and employment systems. Price and Clearihan (20) align with the argument on pressing needs for increased presence of female voice in health leadership context. They discuss women's perceptions of restricting capacity to engage leadership roles focusing on work/life balance in the sense of assuming large amount of domestic work, and on inflexible work environment, such as the inconvenient time and location of professional meetings. The organizationally and socially rooted women's leadership deficit in healthcare was also explored through the lens of perceptions on women's capabilities, credibility and capacity in functioning properly in formal professional roles. Bismark et al. (9) argue that these perceived deficits derive from internalized beliefs about traits and qualities of women who aspire to be leaders. The lack of mentoring, the (un)conscious biases, the male dominated working environment and the conservative social norms in terms of uptake of leading career pathways while running a household have also been hold responsible for the sturdiness of perceptions on women's leadership deficit (49).

Within Greek healthcare context, the described organizational barriers, such as reinforced stereotypes, inflexible work environment, may be considered to reflect dynamic, overlapping, and cross cutting relationships amongst organizations, individuals and socially constructed perceptions about women and leadership. Those approaches were also supported by the comments made by two respondents who emphasized deep-rooted stereotypes and lack of organizational support, confirming Claus' argument that the durability and transferability of gendered perceptions amongst cultures and groups of individuals are difficult to eradicate (2013). Gendered asymmetries in healthcare may contribute to perpetuation of stereotypes hinting the pathway to cultural reproduction of male dominance in professional settings (34). Nonetheless the high social regard of health professions, organizational and cultural mechanisms may explain the underlying interactions between gender and the choices and barriers related to gendered professional careers in health (50, 51). On top of that, the dramatic suffrage of healthcare sector (28) resulted in employment contracts' derailment (29) affecting predominately women and single parent families in health, care and education sectors (31). The imposed social, economic, and organizational constraints may have burden women's perceptions on barriers to their career advancement; thereby, complex relationships between the labor market, gender norms, and economic instability may have interplayed to limit women's choices and possibilities both within professional and social settings (52).

Gendered barriers to equal opportunities in career advancement within working environments may be considered to be the product of dynamic relationships between individuals, organizations, and society. They cannot and should not have an absolute character and need to be subjected to assessment overtime and within professional and socio-cultural contexts. Diversity and inclusion are essential to promote cultural awareness and change, challenge conventional ideas, and improve performance across organizations (53). Perceived leadership abilities and positions correlate closely with gender representation within healthcare sector; the health profession is still highly perceived socially (51) and may assume the role of change agent in ongoing transformation toward sustainability of healthcare sector and societies as well. Therefore, the talent pipeline for women healthcare leadership needs to be supported and enhanced (54–56).

Identifying perceptions on barriers that may hinder the development of women potential may be an essential first step for further evidence base research on the reasons and potential solutions to address gendered challenges in health workforce. Acknowledging the complexity of the phenomenon, the challenge is to explore further these barriers and acquire in depth understanding of involved actors and context; barriers through contextual lens may be framed as an opportunity to develop evidence informed strategies and policies in education and employment and promote inclusiveness and sustainability in healthcare organizations and modern societies. Besides, our task is to seek out and grow leaders, women and men, able to pave the way toward an inclusive and sustainable transformation of healthcare sector and society as well (57).

### Limitations

This study explored the perceptions of the relative importance of barriers to women's leadership in healthcare in Greece. The researchers recognize the limited number of participants in online survey even though the sample size was deemed appropriate for the study's exploratory purposes.

The researchers acknowledge the women leaders' perceptions to be initial and exploratory; they are not considered to contribute in obtaining population based outcomes. However, comprehensiveness of the barriers coding scheme and sampling from all facets of healthcare achieved to provide adequate initial descriptions of women leaders' perceptions on barriers. Hence, the exploratory findings of organizational and cultural contexts as two major barriers to women leadership may provide orientation for an in-depth country specific research.

In depth qualitative and quantitative research in countries with similar and/or different socio-economic status could yield valuable data to triangulate the findings and provide grounded conclusions on this researched topic.

Due to practical issues, fourteen choice sets were incorporated, while Sawtooth survey was initially designed to include sixteen.

## Conclusion

This exploratory study reports on the perceptions of women leaders of barriers in pursuing leading positions within Greek healthcare context. The findings point mainly to organizational and socio-cultural related barriers potentially aggravated by country's unfortunate current economic turbulence. Further extensive research on perceptions of women and men is required to establish grounded conclusions and better inform education and policy makers in developing gender sensitive strategies to sustainable health workforce development.

## Ethics Statement

Research conducted according to ethical principles. Ethical approval was received from Ethics Committees from Maastricht University (No METC 16–4-266, January 19, 2017) and National and Kapodistrian University of Athens (Medical School) (February 3, 2017).

## Author Contributions

SK and KC were involved in the conception of the study. SK KC, MH and KLC were involved in the design of the study. MH conducted the data analysis. Data interpretation was carried out by SK and KC with input from MH and KLC. SK and KC drafted the manuscript with input from MH, KLC and SB. All authors approve the final version of this manuscript and all authors agree to be accountable for all aspects of the work.

### Conflict of Interest Statement

The authors declare that the research was conducted in the absence of any commercial or financial relationships that could be construed as a potential conflict of interest.
